# Botulinum Toxin and Dynamic Splint Restore Grasping Function after Stroke: A Case Report

**DOI:** 10.3390/ijerph20064873

**Published:** 2023-03-10

**Authors:** Denis Moskiewicz, Małgorzata Mraz, Dagmara Chamela-Bilińska

**Affiliations:** 1 Department of Physiotherapy, Wroclaw University of Health and Sport Sciences, 51-612 Wrocław, Poland; 2 Rehabilitation Department, T. Marciniak Lower Silesian Specialist Hospital, Emergency Medicine Center, 54-049 Wrocław, Poland

**Keywords:** rehabilitation, grip, hand, orthosis, SaeboFlex, spasticity

## Abstract

Evidence on the effectiveness of upper extremity rehabilitation post-stroke is inconclusive. We evaluated a tailored therapeutic program with dynamic splint and botulinum toxin injections for the treatment of upper extremity muscle spasticity. A case of a 43-year-old woman with chronic spastic hemiparesis after ischemic stroke with significant mobility impairment in the left upper extremity was described. A 16-week program consisted of three 50-min sessions daily and focused on grasping and releasing with and without the splint. The patient was evaluated before botulinum toxin injection and after 6, 12 and 16 weeks according to the International Classification of Functioning, Disability and Health, and included the following scales: Fugl-Meyer Upper Extremity Assessment (FMA-UE), Modified Ashworth Scale, Numerical Rating Scale (NRS), MyotonPro, Stroke Impact Scale, Box and Blocks. Photographic documentation made before and after the experiment was compared. Motor functions improved by 19.7% on FMA-UE, spasticity was reduced by one degree and pain at rest and during activity decreased by one score on NRS. A reduction in the oscillation frequency of the relaxed muscle and the stiffness of the examined muscles was observed. The patient regained grasping function. Health-related quality of life was systematically improving with a 35% increase at week 16 compared to the baseline. The combination treatment for spasticity based on botulinum toxin and SaeboFlex^®^ dynamic splint in a patient with chronic spastic hemiparesis reduces disability and improves quality of life. However, further research is needed to investigate the treatment results.

## 1. Introduction

People with spastic hemiparesis after a stroke experience functional limitations on many levels. Limited daily activity, primarily due to spasticity, is the most common complication [1], which determines the patient’s ability to live independently [2]. Regaining the grasping function of the hand is a very difficult goal to achieve, but obtaining even the slightest improvement can be very helpful in restoring the patient’s independence [3]. For humans, grasping is so important that the hand has gained the name of the grasping organ in some anthropology and evolutionary sciences [4,5]. In a large study, Nakajeme et al. showed that only 5% of patients after a stroke regain the functionality of the upper limb; however, after 3 months still as many as 20% do not make any movements. Spasticity of the upper extremity contributes to difficulties in dressing, maintaining hygiene and reduces independence in everyday activities by disturbing the grasping function [6]. The recommended first-line treatment in local post-stroke spasticity is the injection of botulinum toxin (BoNT) [7]. Guidelines recommend multidisciplinary rehabilitation after BoNT injections; however, the evidence supporting the effectiveness of such rehabilitation is inconclusive [8]. The limited effectiveness of the therapies used so far results in the constant search and testing of innovative approaches to reduce the level of disability and improve the functional efficiency of patients after stroke [9].

One of the solutions for people after a stroke with moderate and severe disability of the upper extremity is the SaeboFlex^®^ dynamic splint (Saebo, Charlotte, NC, USA). In a process called “agonist retraining”, the patient uses resistance on the fingers and thumb by using the extension spring system. This makes it possible to voluntarily contract and relax the finger flexor muscles, which results in an assisted, functional grip and release [10]. Multiple repetitions of grasping and releasing activities improve volitional motor control of the finger flexor muscles [11,12,13,14]. Pooyania et al. [15] compared the effects of the combined use of the SaeboFlex^®^ dynamic splint and BoNT injections to the use of the splint only in patients with spastic post-stroke hemiparesis. In their study, some patients achieved a functional improvement of the upper extremity, likely due to functional improvement resulting from using the splint for repeated grasp-release training. However, it was not possible to demonstrate a more beneficial effect of using the splint after BoNT administration, so further research was recommended.

A review of the literature encourages using BoNT and the SaeboFlex^®^ dynamic splint in rehabilitation programs to improve functional outcomes in people with chronic spastic paresis of the upper extremity. Thus, we described the case of a woman with local post-stroke spasticity of the upper extremity and evaluated the use of BoNT and the SaeboFlex^®^ dynamic splint in the tailored rehabilitation program for chronic post-stroke spastic hemiparesis. We assumed that a program based on repeated grasp-release activities and BoNT effect will improve the grasping function of the hand.

The aim of this study is to evaluate of the use of BoNT in the treatment of local post-stroke spasticity of the upper extremity in combination with an individual rehabilitation program based on the use of the SaeboFlex^®^ dynamic splint. This research addressed the following research questions:

- Will the applied treatment and rehabilitation program using the SaeboFlex^®^ dynamic splint improve the motor functions of the upper extremity based on the Fugl-Meyer Upper Extremity Assessment scale?
- Will local treatment of spasticity with BoNT and exercise with SaeboFlex reduce pain? Will the applied treatment and rehabilitation program with the use of the SaeboFlex orthosis affect the normalization of torso muscle tone?
- Is it possible to achieve self-grip following local treatment of spasticity with BoNT and exercise with the SaeboFlex^®^ dynamic splint?

## 2. Case Description

### 2.1. Patient

In 2020, a 42-year-old woman had an ischemic stroke in the right hemisphere of the brain, caused by thromboembolic material in the right middle artery of the brain. Before the stroke, the patient was not treated for any chronic disease; she regularly participated in periodic examinations required in the workplace. The factor that could affect the outcome was that despite the rapid thrombolysis (up to 1 h after the first symptoms of stroke), it was not possible to remove the thromboembolic material from the right middle cerebral artery, which resulted in mechanical thrombectomy. Due to imminent cerebral edema, she was inducted into temporary analgosedation. At discharge from the neurology department (on day 29 after the stroke), the patient was conscious, in logical contact, with the presence of a moderately severe syndrome of hemispatial neglect, using a wheelchair and requiring assistance in changing places (based on the neuropsychology consultation). The functional status according to the modified Barthel Index [16] was 30/100. Profound left hemiparesis with a predominance in the upper extremity and pathologically increased muscle tone was observed (internal rotators of the shoulder joint MAS = 1, elbow extensors MAS = 1, elbow flexors MAS = 1+, wrist flexors MAS = 1, finger flexors MAS = 1). Spasticity is caused by caused by damage to the upper motor neuron [17,18,19]. As such, the patient was admitted to the Rehabilitation Department of the T. Marciniak Lower Silesian Specialist Hospital, Emergency Medicine Center, Wrocław, Poland, where comprehensive therapy was applied. It included physiotherapy, occupational therapy and therapy with a neuropsychologist and a neurologopedist.

After a 12-week stay in the rehabilitation department, the patient gained independence to walk on a flat surface and up the stairs. The patient still had paresis of the left upper extremity with spastic muscle tone, without grasping function and with an incomplete range of motion in the peripheral joints (shoulder joint, elbow joint, wrist joint). The functional status according to the Barthel Index was 80/100.

### 2.2. Screening and Justification of the Selection of a Research Participant

The patient was selected with the intention of gaining knowledge about the effectiveness of rehabilitation using the SaeboFlex^®^ dynamic splint in combination with BoNT treatment of upper extremity muscle spasticity in a comprehensively documented manner. There was no expectation of the magnitude of the improvement achieved. The experimental procedures were approved by the Senate Committee for Research Ethics at Academy of Physical Education in Wrocław and comply with the standards of the Declaration of Helsinki. The patient signed informed consent to participate in the study.

The patient met the criteria for the application of BoNT and the use of the splint. For botulinum toxin [20] therapy, the patient was qualified based on the assessment of post-stroke spasticity according to the Modified Ashworth Scale (MAS); a score was ≥2 in at least one muscle group. For therapy with the SaeboFlex^®^ dynamic splint (Saebo, Charlotte, NC, USA), the patient was qualified according to manufacturer recommendations [21], which required having a passive range of 15° of extension movement in the radiocarpal joint with maintaining extension in the finger joints, ¼ of a range of active flexion in the finger joints, ¼ of a range of active foreflexion in the shoulder joint and ¼ of a range of active flexion in the elbow joint. The Mini-Mental State Examination score was 27 points. The patient was aware of her motor deficit, which consisted mainly of a lack of grasping function. She was strongly motivated to participate in the program, had family support, and did not have medical complications. The patient walked on her own, but at longer distances, she used a cane for support. All those conditions made it possible for her to participate in the program.

### 2.3. Methods

**Figure 1:**
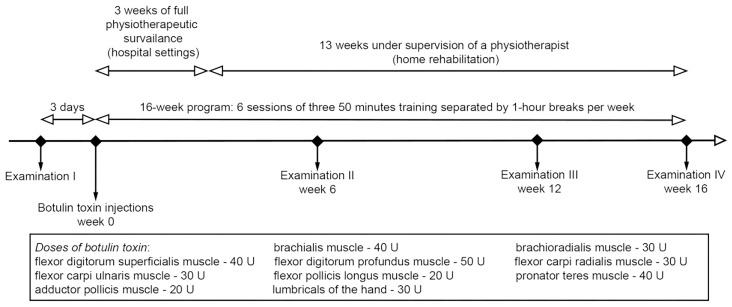
Study design. Created in Photoshop release 24.1.1 (Adobe, San Jose, CA, USA).

The study design excluded the possibility of bias and conflicts of interest. The research was carried out by four physiotherapists who were not the authors of the research-therapeutic program. Their experience should exclude interpretation of the test results’ bias. BoNT was administered by a neurologist at multiple points (except for small muscles) in dose ranges consistent with the summary of product characteristics and with the published recommendations of the Polish Neurological Society expert group. The study design is presented in Figure 1.

### 2.4. Tests and Measures

The tests and measurements were selected to assess the patient’s functional state in agreement with the International Classification of Functioning, Disability, and Health [22]. This classification includes the following subsequent components: functions/structure, activity and participation, thanks to which, it is possible to analyze the results of the applied intervention not only in the context of improving the grasping function of the hand, but also its impact on disability and social participation.

#### 2.4.1. Functions/Structure

1. Level of motor functions of the upper extremity affected by spastic paresis was described using the Fugl-Meyer Upper Extremity Assessment (FMA-UE) [23,24].
2. Assessment of the spasticity of selected muscle groups was performed using MAS [25].
3. Pain sensation was scored on a standard Numerical Rating Scale (NRS) [26] from 0 (no pain) to 10 (worst pain imaginable), at rest and during the activity of reaching for an object placed at a height of 60 cm above the floor (patient in a standing position).
4. Recording of natural muscle oscillations with the MyotonPRO 2013 device [27] (Myoton AS, Tallinn, Estonia) included an acceleration signal and determination of voltage state parameters, as well as biomechanical and viscoelastic properties of soft tissues. The following muscles were assessed: biceps brachii, latissimus dorsi, trapezius pars descendens, flexor carpi radialis, flexor pollicis longus, flexor carpi ulnaris, flexor digitorum superficialis and pronator teres. Measured with MyotonPro, the oscillation frequency f [Hz] of a muscle in a relaxed state (at rest, without volitional stimulation of the muscle) indicates intramuscular pressure and resting muscle tone. At very high f values for a given muscle, blood flow in the muscles may be limited, which may result in, for e.g., faster fatigue. Stiffness is a biomechanical property of a tissue that represents the resistance of a muscle (tissue) to contraction or to a deformation force s [N/m], in this case, to the force with which the MyotonPro probe hits/presses against the muscle [28].

#### 2.4.2. Activity and Participation

1. Stroke Impact Scale (SIS) version 3.0 [29] was used to determine health-related quality of life (test/re-test reliability was 0.79 for general health return after stroke and 0.98 for cognition) [30].
2. Unilateral dexterity and coordination of fine motor skills were checked using the Box and Block Test [31]. The test enables assessing functional grip and its use in the activity. The test uses a wooden box, divided by a partition into two chambers and 150 blocks of the same size. The patient’s task is to move the maximum number of blocks one by one from one compartment of the box to another within 60 s.

#### 2.4.3. Visual Assessment

Photographic documentation and visual assessment were used to evaluate the degree of severity of the associated reactions of the upper extremity while maintaining the standing position (after 20 s of maintaining the position); it is the test at the beginning of the experiment and after its completion (examination I and IV).

#### 2.4.4. Minimal Clinically Important Difference

Minimal clinically important difference (MCID) was considered for FMA-UE, NRS, SIS and MAS scales [32,33,34,35].

### 2.5. Therapeutic Intervention

**Figure 2:**
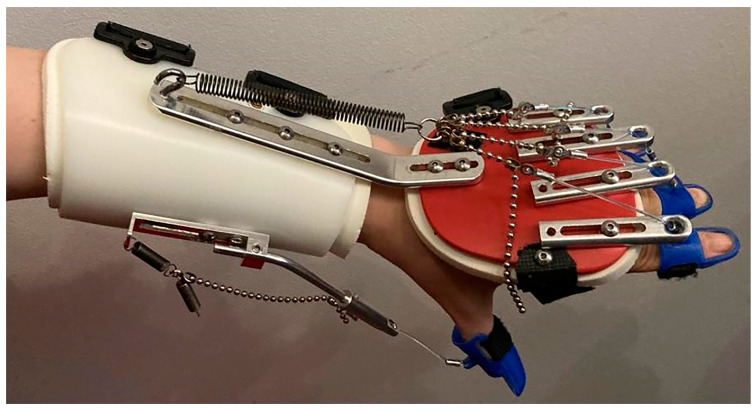
Patient’s upper extremity in the SaeboFlex^®^ dynamic splint.

A therapeutic program with SaeboFlex^®^ dynamic splint according to Butler [36] was conducted by a physiotherapist. The 16-week program focused on grasping and releasing exercises with and without the splint (Figure 2) [37]. The SaeboFlex^®^ dynamic splint consists of a part that includes the forearm shell and a dorsal hand piece which is a platform that stabilizes the palm attached to it. Digit caps for individual fingers from II to V and the thumb are attached to the dorsal hand piece. The individual digit caps are attached to a line made of a durable polymer that provides assistance during extension movement of the fingers. The splint is additionally stabilized with Velcro [38]. The arrangement of springs can be adapted to the current abilities of the patient. The patient used the orthosis for about 2 h a day (75% of the therapy). The splint was used by the patient only as part of exercise; therefore, she did not use it for other activities. We used the SaeboFlex^®^ dynamic splint because it is the only splint available on the Polish rehabilitation market. Other researchers confirm the high quality and effectiveness of this device [10,37,39].

Exercise program according to Butler [36]:

1. Repeated exercises for a single muscle group using the splint (e.g., biceps brachii during lifting the hand towards the mouth); improvement of strength and neuromuscular control in a single muscle group, through the use of concentric, isometric and eccentric muscle contraction.
2. Repetitive exercises involving multiple muscle groups using the splint (e.g., pectoralis major/triceps brachii during reaching and retrieving the upper extremity); improvement of the strength and neuromuscular control of agonist and antagonist muscles through concentric, isometric and eccentric muscle contraction. Reaching exercises were carried out both in sitting and standing positions. The patient’s task was to reach for an object (a ball) on the table and put it in another place. The height of the table was variable, which was aimed to diversify the activity. Recreation/creation of new movement engrams by repeating the same tasks.
3. Repetitive grasp-release exercises of objects using the splint; improvement of volitional motor control of the finger flexor muscles through the use of a phenomenon called “agonist retraining”. The use of resistance imposed by a spring system on the fingers and thumb, which results in the patient performing voluntary muscle contraction and relaxation of the flexor muscles of the fingers and thumb. During the exercises, the type of objects and their location were changed.
4. Repetitive grasp-release exercises without the splint; objective: to consolidate the upper limb functions obtained with the splint by repeated grasping and releasing tasks of various objects placed in different locations.

The total number of repetitions of all exercises was approximately 400–600 as such a number of repetitions of difficult functional tasks such as grasping during daily activities can lead to brain reorganization [40]. Experimental models on animals have shown that 400–600 repetitions per day are needed to provoke changes in the nervous system [41]. Task form exercises are the most effective as they require the patient to concentrate and make an effort to reach or grasp objects. The task should not be too difficult, but it should not be too easy—it should be a challenge for the patient. Research suggests that intensive task-based training with a large number of repetitions improves the upper extremity function [42]. Research on neuroplasticity has shown that repeated repetition of tasks can affect changes in cortical organization [43].

## 3. Results

Examinations II to IV showed an improvement in motor function compared to examination I. After completion of the program, motor function of the upper extremity increased by 19.7%, pain sensation by 8.3%, and passive range of motion by 16.7%, while arthralgia decreased by 12.5%. Changes in the wrist and hand motor skills as well as coordination/velocity were noticed only during examinations III and IV. Sensation changed only slightly during examination IV, while arthralgia decreased gradually starting from examination II.

Spasticity was reduced by one score on average in all the muscle groups. The greatest change in spasticity was observed during examination II, which resulted from the effect of BoNT.

Pain at rest and during activity decreased only by one score but remained at a fairly high level.

**Figure 3:**
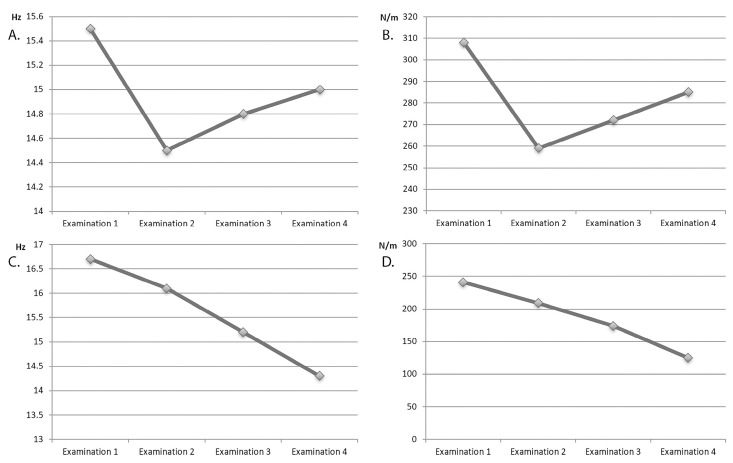
The direction of characteristic changes in muscle tone of selected muscles of the upper extremity and trunk in a standing position during examinations I to IV. (**A**). Oscillation frequency of flexor digitorum superficialis. (**B**). Stiffness of flexor digitorum superficialis. (**C**). Oscillation frequency of latissimus dorsi. (**D**). Stiffness of latissimus dorsi.

Oscillation frequency and stiffness gradually decreased starting from examination II (Figure 3). A particular drop was observed during the active treatment with BoNT (muscles 1–6, examination II). The trunk muscles (muscles 7–10) were not subjected to treatment with BoNT but responded to treatment during examinations II to IV.

The total SIS score increased by 20%, while the HRQoL-physical domain improved by 15%. Dexterity and coordination of fine motor skills changed during the study. During examinations I and II, there were no fine motor skills. The grasping function was achieved after 12 weeks of therapy and improved further at 16 weeks. The results are presented in Table 1.

The MCID of 18.8% for FMA-UE met by the patient as the difference between examination I and IV was 19.7%. The MCID of one score for MAS was met as such a difference and was achieved for three of five muscle groups. For NSR, MCID is considered to be a one score or 15% difference, which was achieved by the patient both at rest and during activity. For the SIS questionnaire, the MCID values of 9.2 for strength, 5.9 for ADL/IADL, and 4.5 for mobility were met. For the hand function, the MCID was close to the reference value of 17.8 as the difference achieved was 16 (Table 1).

**Figure 4:**
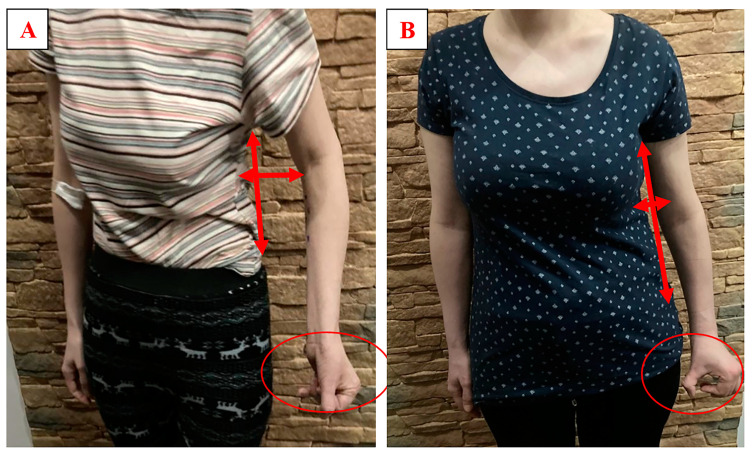
Patient before treatment for spasticity with botulinum toxin (**A**) and the patient after a 16-week tailored therapeutic program using the SaeboFlex^®^ dynamic splint combined treatment for spasticity with botulinum toxin (**B**). Vertical arrow shows the difference in the torso: shortened trunk (**A**) vs. elongated trunk (**B**). Horizontal arrow shows the distance between the trunk and the upper limb: large distance (**A**) vs. distance reduced (**B**). No side effects were observed.

The visual assessment (Figure 4) of the severity of the associated reactions of the patient’s upper extremity at a standing position showed a significant change in the position of the upper extremity. It was positioned closer to the patient’s body (increased adduction and external rotation of the shoulder joint). The elbow and finger joints showed a smaller flexion position together with the elongation of the patient’s trunk on the left side.

## 4. Discussion

According to the literature, the presented case report and the experiment used are innovative, and its effects encourage the use of such a therapy program in a larger group of stroke patients with spastic paresis of the upper extremity. The effects of the experiment were evaluated according to ICF standards, which is a strength of this report. In our case report, we showed that a 16-week program using BoNT treatment for spasticity and exercises with dynamic splint improved functional grip and release. Furthermore, it improved the functional status of a patient with chronic, moderate mobility impairment resulting from stroke caused by the presence of thromboembolic material in the right middle artery of the brain.

Physiotherapy for post-stroke patients aimed at recovering lost functions appears to be the most effective form of neurological rehabilitation [44]. Its main goal is to regain as much independence as possible in everyday activities, resulting in an improvement in HRQoL. Continuously growing knowledge of post-stroke rehabilitation confirms its central role. Obtaining more evidence for the effectiveness of rehabilitation of post-stroke patients and facilitating the transition to clinical practice requires a better understanding of the neurophysiological mechanisms that support recovery after stroke and the impact of physiotherapeutic interventions on these mechanisms.

Physiotherapeutic practice shows that post-stroke rehabilitation focuses mainly on gait re-education, while restoring/improving the function of the upper extremity is often overlooked. Lang et al. showed that during a single therapeutic session, the most frequently practiced activity is walking with 357 steps on average [45]. On the other hand, Kimberley et al. depicted that the average number of repetitions of activity for the upper extremity during a single treatment session is only 23–32 [41]. These show the imperfections of the therapeutic process and also certain habits of the therapists. Therefore, we proposed a program that can be beneficial in setting the goals for therapy.

Wade proved that spasticity is a common complication after stroke that limits voluntary movements [46], which can lead to reduced effectiveness of rehabilitation. Nasb et al. demonstrated the high effectiveness of combination therapies in reducing spasticity associated with the return of motor functions in the upper extremity [47]. The authors compared the effects of constrain-induced movement therapy combined with BoNT to intensive conventional therapy also combined with BoNT in patients with partial, active extension in the wrist, finger and thumb joints. Both modalities improved motor functioning and activities of daily living in post-stroke patients.

The evidence on the efficacy of combination treatments with BoNT in patients with greater motor deficits is limited. To meet the needs of these patients, we developed a 16-week program in which the SaeboFlex^®^ dynamic splint and BoNT are used. Previously, Pooyania et al. [15] showed a 12% improvement in motor function in patients in the chronic period after stroke treated with SaeboFlex^®^ dynamic splint and BoNT. Our results are in line with this study; however, the improvement in FMA UE was greater, by 19.7%, and highlights the benefits of the tailored program. To our knowledge, the program is novel and encourages its use in a larger group of post-stroke people with spastic paresis of the upper extremity, particularly due to its compliance with the standards of the International Classification of Functioning, Disability, and Health.

Our report showed favorable changes in the functional status of the patient. The final result of the FMA UE test showed that the patient achieved more than 50% of the motor function of the upper extremity affected by spastic paresis. This translates into a 20% improvement in impaired motor function of the entire extremity. Although the dynamic splint stimulates grasping and releasing movements, a favorable change was observed in other joints, i.e., in the elbow and shoulder joints. This confirms that the performance of the grasping task stimulated by the splint was conducted as a functional model of the extremity and as such serves as an example of a motor learning principle applied in therapy, which is based on intensity, task, variability, increasing difficulty, goal orientation and a large number of repetitions. The principles of motor learning stimulation in the treatment of stroke patients were presented by Maier et al. [48]. Thus, the proposed program meets the principles of therapy based on stimulating motor learning. This effect is also confirmed by the Box and Blocks test result, which showed the return of independent grasping function already after 12 weeks of therapy and further functional grip improvement after 16 weeks of therapy.

Regaining the grasping function of the hand is a very difficult goal to achieve; however, obtaining even the slightest improvement can be very helpful in restoring the patient’s independence [3]. According to the guidelines for treating spasticity with BoNT described by Sławek, the peak of BoNT activity occurs in the second week after injection, while after 12 weeks, the effect of the drug wears off [49]. We considered this effect and confirmed it in practice. Thus, during examination II, the resolution of spasticity was observed together with the highest changes in oscillation frequency and muscle stiffness. Despite the fact that after 12 weeks, the effect of BoNT ceased, muscle tone was lower than before the experiment. This should be considered a beneficial effect of the applied therapy in reducing the level of spasticity and regulating muscle tone.

Park and Chung reviewed the literature to summarize the effectiveness of treating neuropathic pain with BoNT [50]. They demonstrated that BoNT is effective and reduces pain in the shoulder joint in patients after stroke. However, this effect was not observed in the presented case. No significant reduction in pain was achieved, both as a result of BoNT (examination II) and in the following weeks of therapy.

The improvement in the Box and Blocks test result is very important. After 6 weeks, the patient still showed no grasping function. However, after 12 weeks, the patient obtained a functional grip, which improved by 100% after 16 weeks. These results suggest a beneficial effect of the program on the grasping function of the hand, but the time needed for improvement must be longer (12 weeks). Furthermore, a noticeable further improvement was visible at a time when BoNT was no longer active.

The muscle tone of the trunk muscles in the supine and standing positions showed improvement at each subsequent measurement. From examinations II to IV, the oscillation frequency and muscle stiffness decreased. This indicates the normalization of trunk muscle tone as a result of the gradual change in the patient’s postural compensation strategies.

The activity of the trunk muscles during reaching movement was described by Marchesi et al. [51]. They showed that reaching movement requires the employment of various kinematic strategies. The greatest activation was exerted by the latissimus dorsi muscle and obliquus externus abdominis muscles. Cabanas-Valdes et al. proved that additional training of the trunk muscles improves core control, balance in sitting and standing positions, walking and performing activities of everyday life in patients in the subacute phase after stroke [52]. Proper core stability and proper control of the trunk muscles are fundamental for most daily activities such as standing up, sitting down, walking and stabilizing the distal parts of the extremities. Dean et al. demonstrated that both of these factors are necessary to maintain a stable posture and shift body weight when performing activities such as reaching movements [53]. The authors observed that depending on the reaching distance, there a relationship appears between the movement of the trunk and arm, the tone of the lower extremities and the increase in the activity of individual segments of the trunk and feet. Bending the trunk to extend the range increases the postural requirements because the position of the center of gravity of the body changes during reaching movements.

Chern et al. showed that repeated training with a variable velocity which consisted of bending and reaching for objects located in different places can stimulate the activity of postural control systems [54]. The authors compared the effect of those activities between healthy people and stroke survivors. They showed that the changing location of the target and difficulty level have an impact on the displacement of the center of gravity of the body and are good tools for training postural control.

De Baets et al. showed that correct activation of the trapezius muscle by ascending a part at the beginning of the movement is key for the proper function of the arm [55]. The conclusions of Chern et al. [54] and De Baets et al. [55] are in line with our report, in which muscle tone of the latissimus dorsi and the descending part of the trapezius decreased. Furthermore, changes seen in the visual assessment suggest that improving the stability of the lower extremity support phase is related to reaching movements and grasping objects during exercises with the splint.

Hartley et al. demonstrated that rehabilitation of post-stroke people has a beneficial effect on HRQoL [56]. The authors conclude that psychological support and simultaneous pain treatment are very important in therapy. Our results are in line with these findings as the patient was shown to systematically improve her HRQoL.

In the case of a novel treatment with limited evidence, in addition to effectiveness, the patient’s perspective is important. The patient and her family confirmed improvements in the patient’s functional state and upper extremity function each week as they recovered grasping function. The patient was critical of the duration of exercise both during the day and throughout the experiment. Despite positive effects, the program required commitment and effort.

## 5. Conclusions

The results of the experiment show a positive effect on treating spasticity of the upper extremity with BoNT in combination with a tailored therapeutic program using the SaeboFlex^®^ dynamic splint in a patient after stroke at all levels of the International Classification of Functioning, Disability, and Health. After 16 weeks of therapy, muscle tone normalized as a result of a reduction in spasticity as well as frequency oscillations and muscle stiffness within the upper extremity and trunk. The motor function of the upper extremity improved, and the grasping function was regained. Although pain remained almost at the same level, the patient’s quality of life improved. The positive effects of the treatment translated into the patient’s self-esteem and the effects on her daily functioning were noticed by her family. The combined treatment with thed BoNT and SaeboFlex^®^ dynamic splints can improve functional status in post-stroke patients. The conducted experiment is a pilot study. The result obtained encourages the authors to undertake further research on a well-characterized and fairly homogeneous group of people after a stroke. The results of further research will allow conclusions regarding the population of people post-stroke to be drawn.

## Figures and Tables

**Table 1:** Results of structure/function measurements, oscillation frequency and stiffness of selected muscles during examinations I to IV.

Test	Examination I	Examination II	Examination III	Examination IV
Structure/function measurements				
Fugl-Meyer Upper Extremity Assessment (A–D, max 66)	21/31.8%	24/36.4%	33/50%	34/51.5%
Upper extremity (max 36)	18	21	25	25
Wrist (max 10)	0	0	1	2
Hand (max 14)	3	3	6	6
Coordination/velocity (max 6)	0	0	1	1
Pain sensation (max 12)	8	8	8	9
Passive range of motion (max 24)	12	13	15	16
Arthralgia (max 24)	6	9	9	9
Modified Ashworth Scale				
Elbow flexors	3	1	1+	1+
Elbow extensors	2	0	1	1
Wrist flexors	1	0	1	1+
Finger flexors	2	1	1	1+
Thumb flexors	2	1	1	1
Numerical Rating Scale				
At rest (max 10)	8	7	7	7
During activity (max 10)	9	8	8	8
Oscillation frequency [Hz] and stiffness [N/m] of selected muscles				
1. Pronator teres				
Oscillation frequency	14	10.7	10.9	11.5
Stiffness	229	119	124	188
2. Flexor carpi ulnaris				
Oscillation frequency	19	15.5	16.5	16.9
Stiffness	403	294	310	329
3. Flexor carpi radialis				
Oscillation frequency	13.5	10.7	12.4	12.6
Stiffness	218	124	190	198
4. Flexor pollicis longus				
Oscillation frequency	17	15.2	16	16.3
Stiffness	316	227	296	301
5. Flexor digitorum superficialis				
Oscillation frequency	15.5	14.5	14.8	15
Stiffness	308	259	272	285
6. Biceps brachii				
Oscillation frequency	16.2	14.8	15.2	15.5
Stiffness	295	259	265	270
7. Latissimus dorsi				
Oscillation frequency	15.8	14.4	13.5	12.4
Stiffness	305	249	199	129
8. Trapezius, descending part				
Oscillation frequency	15.5	13.6	12.9	12.8
Stiffness	291	247	240	251
9. Latissimus dorsi (standing position)				
Oscillation frequency	16.7	16.1	15.2	14.3
Stiffness	241	209	174	125
10. Trapezius, descending part (standing position)				
Oscillation frequency	19	18.3	18.3	17.9
Stiffness	375	352	328	377
Activity and participation				
Stroke Impact Scale (0–100%)				
Strength	40	40	48	52
Memory and thinking	62	91	91	91
Emotions	42	71	71	76
Communication	86	94	97	97
ADL/IADL	54	70	74	76
Mobility	78	82	82	84
Hand function	24	32	36	40
Role and social function	40	43	58	65
Physical domain (1, 5, 6, 7)	56	64	68	71
Total (1–8)	57	69	73	77
Box and Block Test	0	0	1	2

ADL/IADL, activities of daily living/instrumental activities of daily living.

## Data Availability

Data available from the corresponding author on request.
